# Thinking and Acting with School Children in Fukushima: Implementation of a Participatory Theater Approach and Analysis of the Experiences of Teachers

**DOI:** 10.31662/jmaj.2019-0031

**Published:** 2019-11-08

**Authors:** Aya Goto, Alison Lloyd Williams, Yujiro Kuroda, Kenichi Satoh

**Affiliations:** 1 Center for Integrated Science and Humanities, Fukushima Medical University, Fukushima, Japan; 2 Department of Sociology, Lancaster University, Lancaster, UK; 3 The Center for Data Science Education and Research, Shiga University, Shiga, Japan

**Keywords:** Children, school teachers, Fukushima nuclear accident, humanities, arts, community networks, resilience

## Background

The Fukushima Nuclear Power Plant accident occurred in 2011, and mothers of young children were identified as one of the groups at risk of negative emotional and mental health consequences ^(1)^. Bromet worked with and followed mothers and children who were affected by the Chernobyl nuclear accident and found that the disaster experience was reflected in mothers’ perceptions of their children’s well-being ^(2), (3)^. However, their anxiety was not transmitted to the children themselves, and a comparison of mental health and teachers’ evaluation did not differ between the affected and matched non-affected children ^(2)^. In the Sendai Framework for Disaster Risk Reduction, it is stated that “Children and youth are agents of change and should be given the space and modalities to contribute to disaster risk reduction, in accordance with legislation, national practice and educational curricula” ^(4)^. Although scientific evidence is accumulating around the consequences of the Fukushima nuclear accident on children’s physical and mental health ^(5), (6)^ and more recently on their social life including bullying ^(7)^, not much has been reported about the roles that children are taking in the disaster restoration.

Research with children affected by the 2013–2014 UK winter floods has shown the active role that young people can play in community recovery and resilience building and the use of arts to promote children’s voices in disaster management ^(8), (9)^. Children’s Flood Manifestos were developed as an outcome from the project, which stated that children should know their local area as part of flood preparedness. Lloyd Williams of the UK project has applied the participatory theater approach in various global settings and has shown ways in which it can enhance children’s role as active citizens in community development and resilience building ^(9), (10)^.

We have adapted Lloyd Williams’s approach to work with Fukushima children within the Japanese education system. The second author (ALW) consulted with the first author (AG) and school teachers to devise the program. The first trial, conducted in 2016, involved a series of workshops with children, spanning over five weeks, leading to a 20-minute public performance. A single 90-minute “mini workshop” based on the larger-scale program was developed and tested with classes of children in 2017 and applied further in the teacher training conducted in 2018 as a way of supporting children to think about their local community. These teachers are the key players of the school education system, and the importance of their roles has been acknowledged since the Fukushima nuclear accident ^(11)^. Our workshop was designed and conducted in collaboration with school principals to ensure the acceptance and sustainability of our innovative theater-based trial. The present study aims to visualize the experiences of the teachers who attended the training by using the text-mining method.

## Methods

The program took place at public elementary schools in Date City in Fukushima involving the same school principal throughout to ensure smooth implementation. Table 1 shows the content of the “mini workshop”. The introductory and warm-up sections helped students start feeling comfortable about expressing through their body and voice and begin working collaboratively. In the main section, the students discussed aspects of their community and expressed their opinions through the creation of short scenes to be performed to their classmates. The participating groups of children comprised 6^th^ graders in 2016, 4^th^ and 5^th^ graders in 2017, and 5^th^ and 6^th^ graders in 2018. The selection of student groups depended on the schedules of the schools and research teams. In 2018, the school principal expanded the program to an “open teacher training” and invited nearby school teachers. The teachers participated in a children’s workshop, followed by a seminar about the participatory theater methodology. Six teachers attended the training, including the teacher whose students participated in the original 2016 program and two whose classes piloted the 2017 “mini workshop.” The ethics committee of Lancaster University and Fukushima Medical University approved this study (No. 30157), and written informed consent was collected from teachers.

**Table 1.** Content of the Participatory Theater “Mini Workshop” for Elementary School Students.

Preparation	
·	Open space (gym), large sheets of papers (one per group), and marker pens (different colors)
·	Two facilitators
1. Introduction (10 mins)	
·	Stand in a circle
·	Practice greeting in English (words and handshake)
·	Explain the aims of the session: to share ideas about where we live through discussion and action
2. Warm-up (20 mins)	
·	Active exercises: Singing “London’s Burning” (sing with actions) and “Stop Go Jump Clap” (perform the actions according to prompts)
·	Concentration exercises: “Mirrors” (mirror the actions of the other in pairs)
3. Main activity 1: Thinking about where we live (15 mins)	
·	Students discuss and write in groups what they like about their local community (2017) and what they would like to change about it (2018).
·	Each group presents their favorite ideas and their thought behind it.
Break (10 mins)	
4. Main activity 2: Showing where we live (25 mins)	
·	Groups create short drama scenes based on their favorite idea (approximately 30 seconds long) using actions and sounds and conveying their opinions and attitudes.
·	Groups show their scenes to one another and give feedback on what they saw.
5. Review and closing (10 mins)	
·	Sit back in a circle
·	Invite students to ask any questions they want to ask
·	Sing “London’s Burning” again

The present study focuses on the experiences of the six teachers who participated in the 2018 training and submitted a report about their learning and perceived difficulties in applying the methods. These reports were written in Japanese and translated into English by a bilingual retired local teacher and the first author before beginning the analysis. The text-mining analyses were conducted using KH Coder, version 3 ^(12)^. The frequently used words were listed, and the sub-graph analysis of a co-occurrence network was conducted to classify these words into major topics. The Jaccard coefficients were calculated to determine the edge strength in the co-occurrence network. Those with top 60 strength were drawn in the diagram, and closely associated words were color coded. Correspondence analysis was also used to examine differences in word usage among first-time/repeat attendants and learning/difficulties and to visualize in two dimensions. The relative locations between words and groups show the relative frequencies as in a contingency table. Words that appear on the opposite side of the contrasting group are characteristic of the target group.

## Results

In comparison to the initial 2016 program, the mini workshop was much more concise. However, it still followed the same approach and structure, namely, identifying and exploring ideas on the local community, choosing which aspects to perform, creating scenes in groups and performing to one another, and reflecting on each other’s performances.

**Figure 1.**
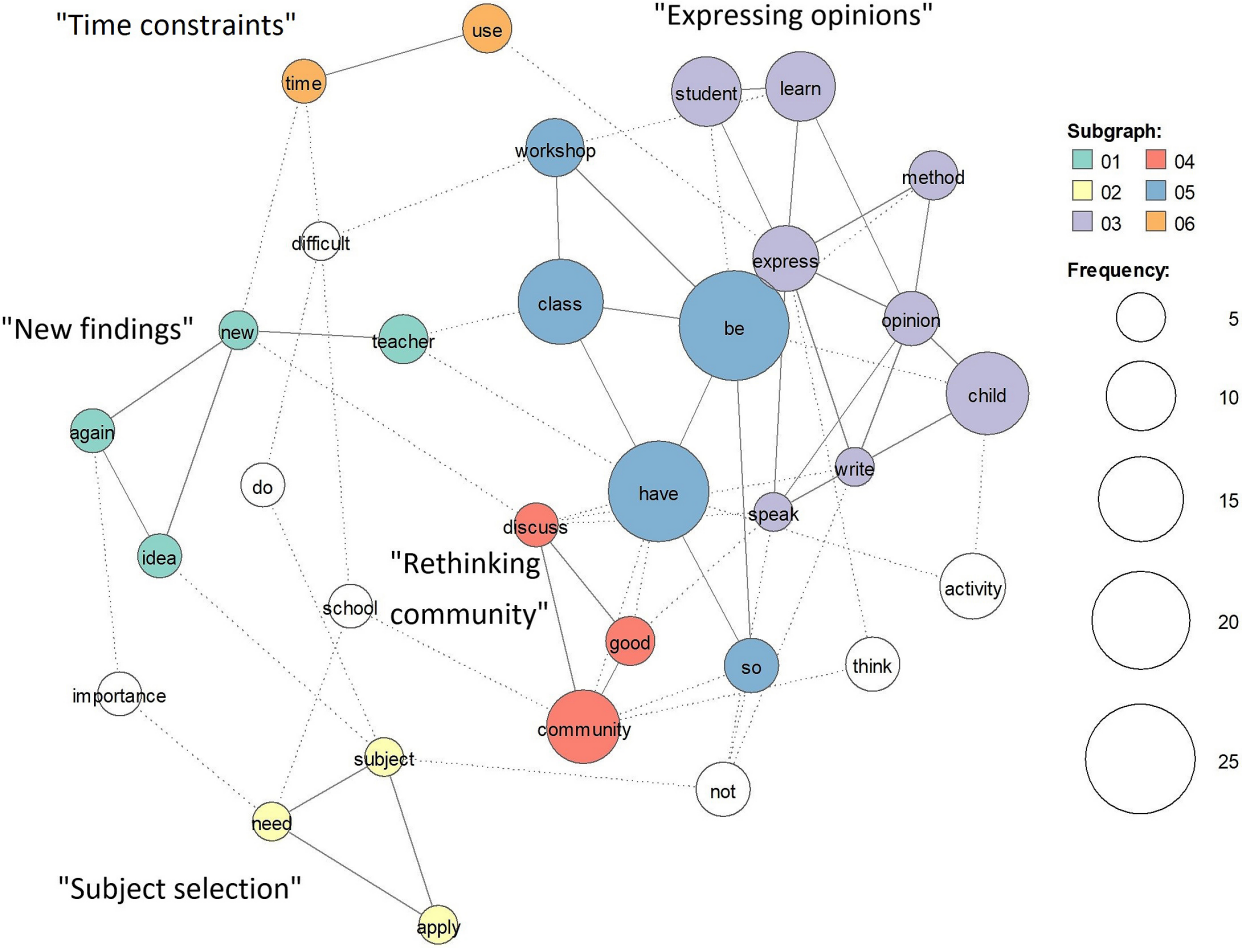
Sub-graph analysis of a co-occurrence network among the words used in the reports of the teachers about the workshop. Each color indicates a cluster of words (topic) used closely in sentences. By referring to the hierarchical cluster analysis, the blue cluster (05) was not considered as one topic because it included auxiliary verbs (be and have) connected with multiple topics. The following five topics were extracted: 01 “new findings,” 02 “subject selection,” 03 “expressing opinions,” 04 “rethinking community,” and 06 “time constraints.”

The total number of sentences written by the six teachers (three first-time attendants and three repeat attendants) was 54 (learnings: n = 40, difficulties: n = 14). The top five frequently used words (limited to verbs, nouns, adjectives, and adverbs, excluding the verbs be and have) were “class” (used 15 times), “child” (used 14 times), “learn” (used 11 times), “community” (used 10 times), and “student” (used 10 times). The sub-graph analysis of a co-occurrence network (Figure 1) among words used three or more times showed five major topics. Of note, by referring to the hierarchical cluster analysis, the blue cluster was not considered as one topic because it included auxiliary verbs (be and have) connected with multiple topics. Three topics (“new findings,” “expressing opinions,” and “rethinking community”) were about learning, and two were about difficulties (“time constraints” and “subject selection”). An example opinion for the “new findings” was “*A new discovery comes out from an unexpectedly small idea…*” (repeat attendant). Another example opinion of the “expressing opinions” was “*Students’ creativity will improve through expressing the environment, objects and people through their bodies.*” (first-time attendant). It was mostly repeat attendants who mentioned “rethinking community,” for instance, “*I learned the importance of reflecting children’s opinions in the community.*” (repeat attendant). Notably, the difficulties expressed by the teachers were mostly about which subject to apply the method and time constraints in the curriculum.

**Figure 2.**
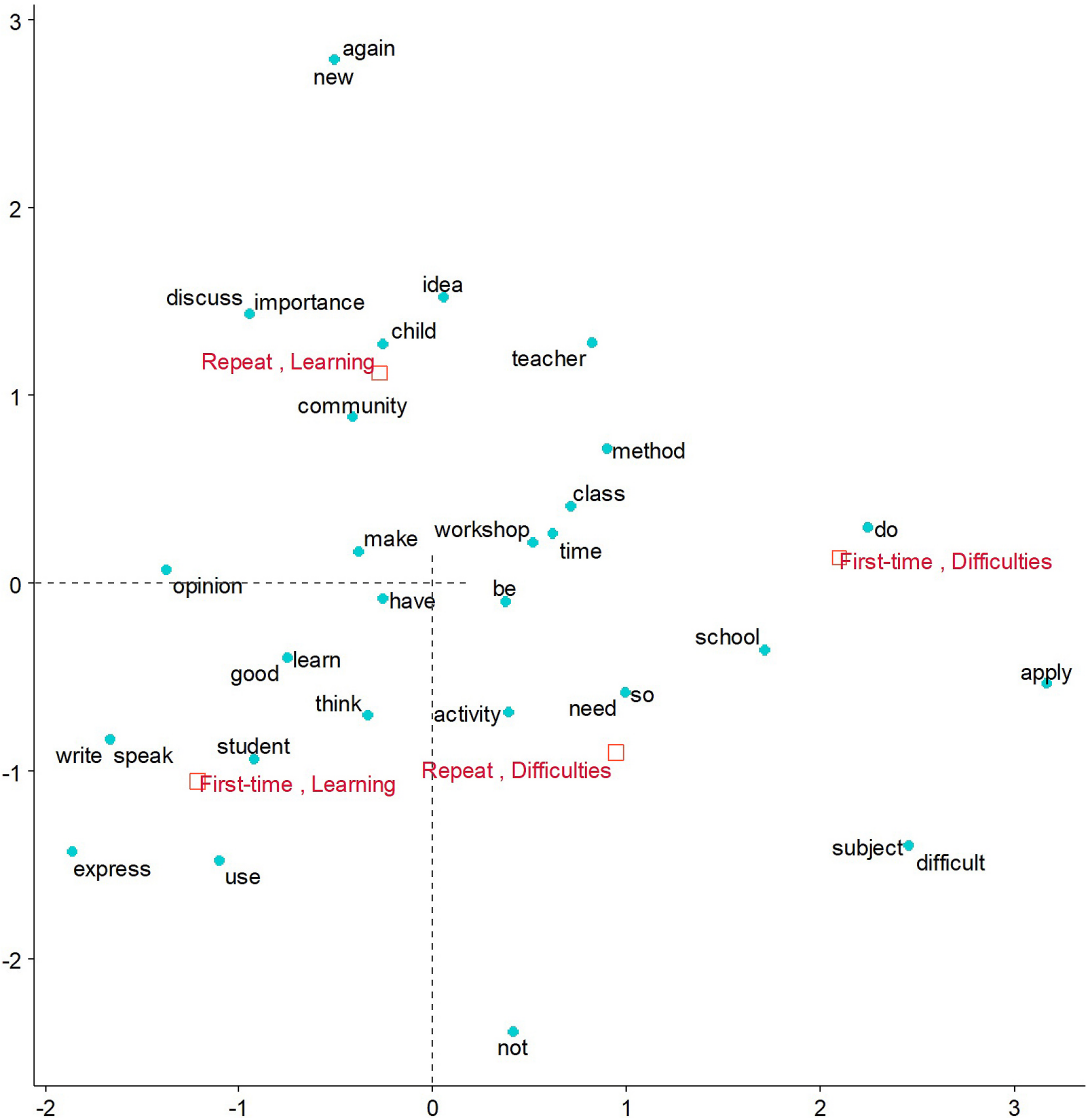
Correspondence analysis of teachers’ opinions about the training: differences by past attendance. A short distance between words indicates that these are used close together in sentences and that between-group labels indicate their similarities. Words that appear on the opposite side of the contrasting group are characteristic of the target group.

The correspondence analysis among the top 50 words used three or more times (Figure 2) showed that the difference between first-time and repeat attendants was more distinct for the learning than difficulties. The first-time attendants voiced that they learned how children can express their opinions through theater, as reflected in an example shown in Figure 1. The repeat attendants mentioned more about technical points and noted students’ self-learning capacity (Figure 2). For instance, they learned the importance of warming up and that of maintaining a supportive, rather than directive, role as they work with the children while responding to their questions when they ask. Additionally, the repeat attendants noted students’ self-learning capacity as follows: “*School tends to dominate children. I realized again the importance of children's independence in learning.*”

## Discussion

The present study describes an innovative trial of adapting and implementing a participatory theater with children in Fukushima and a way to quantitatively visualize teachers’ opinions. The teachers learned how the creativity and capacity of children to express their opinions about community could be enhanced through the methods used in the mini workshop. The repeat attendants became more aware of the importance of the voices of children in community resilience and showed an interest in learning more practical skills for running the workshop. Issues remain around how to incorporate the method into the existing school curriculum in Fukushima.

Reich and Goto describe the need for establishing a system to involve community representatives in restoration activities after the Fukushima nuclear accident by empowering communities and individuals to understand their own situations and decide on actions that are appropriate for them ^(13)^. Although small in study size and with a rigorous evaluation still underway, this trial indicates that a participatory theater could be a way for teachers to work with the next generation to enhance community resilience through valuing children’s creativity and autonomy in expressing issues of concern to them and nurturing teachers’ perspectives to see children as actors in the community. Furthermore, our study showed that the analysis and visualization of text data can provide vivid scientific images of the opinions of the participants and help share experiences of this innovative education project. Similarly, Hasegawa and colleagues reported radiation-related anxieties among Fukushima residents by visualizing the collected text data ^(14)^. The combination of a participatory method and text-data analysis may serve as a model initiative of how to link research and impact in the community ^(15)^.

## Article Information

### Conflicts of Interest

None

### Sources of Funding

This work was supported in part by a JSPS International Post-doctoral Fellowship in Japan FY2016 (Principal investigator: ALW), the Program of the Network-type Joint Usage/Research Center for Radiation Disaster Medical Science 2018 (Principal investigator: ALW), and Triangle Project 2018 (Principal investigator: KS).

### Acknowledgement

The content of the manuscript was presented in part at the Japan Medical Association - Harvard T.H. Chan School of Public Health, Taro Takemi Memorial International Symposium in 2018. The authors thank Ms. Satsuki Katsumi and Mr. Shigeru Nakano (former school principals in Date City) for their contribution towards implementing the program and the teachers and students in Date City for their active participation.

### Author Contributions

AG and ALW designed the workshop, and KS conducted the visualization analysis. AG, ALW, and YK implemented the workshop, and AG and KS analyzed the text data. All the authors were involved in the data interpretation, manuscript writing, and revision and approved the final version.

### Approval by Institutional Review Board (IRB)

The Ethics Committee of Lancaster University (no. unavailable) and Fukushima Medical University approved this study (no. 30157).
